# The New York Homœopathic Union

**Published:** 1897-06

**Authors:** Emma D. Wilcox

**Affiliations:** Secretary


### THE NEW YORK HOMEOPATHIC UNION.

The regular monthly meeting of the New York Homeopathic Union was held at 62 West Forty-ninth Street, New York, Thursday, May 20th, 1897, the President, Edmund Carleton, M. D., in the chair. Members present: Doctors Burd, Clock, Finch, and Jarrett. A number of members sent letters of regret at being detained by important engagements.

The first hour of the meeting was devoted to reading the "Defence of *The Organon*,"* pp. 30-51 inclusive. It was keenly relished, and elicited favorable comment, the following passage for instance:

"Among other things Hecker informs the reader (pp. 72, 73), 'Physicians have given one and several grains of powerful medicines daily to healthy and sick persons, and have often failed to perceive the production of any effects appreciable by the senses.' It is palpably untrue that physicians have hitherto given or seen given to healthy persons even single grains of powerful medicines either daily or quarter-yearly. And in diseases? What may they there have seen? Seeing that the effects of the medicines are mixed up with the symptoms of the diseases, and besides a single, simple, medicinal substance was hardly ever exhibited alone, not even in disease, but in combination with several other drugs. Under such circumstances what could they have seen or observed? Why, nothing at all! What's the object of this silly talk?"

The last hour was spent in relating clinical cases and discussing the same.

Dr. Carleton gave the history of a case of abscesses of both middle ears. Under allopathic management the morbid pro-

---

* See short review of this book in THE HOMEOPATHIC PHYSICIAN for November, 1896, page 488.

cess had gone so far that a specialist had been sent for to operate upon the ears. The drums bulged outward, and felt that way to the patient; ears felt stuffed; deaf; meati swollen; jerking, tearing pain, worse at night; sore mastoids and in front of ears; chilly; thirstless. As Pulsatilla corresponded exactly with these symptoms, it was given, the two-hundredth in water, every two hours. Improvement was noticed the first day, and complete resolution followed quickly. There was no discharge of pus. Hearing was completely restored.

Dr. Jarrett was reminded of a case of abscess of the ear that seemed to demand Silicea. That remedy failed to relieve, and Hepar cured speedily.

Dr. Finch remarked that Silicea was inert in its crude state, and should be given in potency to get results.

Dr. Jarrett enumerated these symptoms: thin, watery discharge, with curds; a hunchback, of tuberculous family; parts sore, could not lie on that side; heavy sweats; coated tongue; offensive breath; no temperature; worse from heat.

Dr. Carleton produced Gross's *Comparative Materia Medica*, which he opened to the comparison between Silicea and Hepar. Members participated in the sifting process. In a few minutes it was demonstrated that Hepar was the nearest similar, by reason of modalities especially.

Dr. Finch verified in a number of cases the aggravation of conium pains in the mammary gland at the menstrual period.

Dr. Clock reported a case that had been pronounced by eminent authority to be scirrhus breast, and condemned to the knife. Staphisagaria cured—the mental symptoms corresponding closely.

Dr. Carleton related his experience with a stubborn case of nocturnal enuresis. Five remedies had been given, after a good deal of study, and each had palliated only, showing that the right medicine had not been selected. The symptoms were meagre. No anatomical obstacle presented. One day the mother of the boy (he was ten years old) sent this word, "Doctor, it smells like cat's urine." Asparagus was found to be the closest remedy having that indication. Hering's *Guiding Symptoms* give the following symptoms of that drug: "Increased urine, beer-brown, without sediment; urine of a peculiar odor; urine of unpleasant odor, second day, N.; urging to urinate—urine of strong smell; urine smells like cat's urine." Asparagus two-hundredth, a powder every night until relieved, wrought a speedy cure.

Dr. Finch had succeeded in curing a troublesome case of nocturnal enuresis with Hydrophobinum, on the indication, "frightened at sound of running water."*

Dr. Burd cured a uterine fibroid with Kali-chlor., twelfth decimal. Patient had lost thirty-one pounds in weight; had oppression of the chest, worse when lying; sleeplessness. The other indications the doctor did not recollect.

Also a mammary gland that had been called cancerous by a number of physicians. Patient was dizzy, especially when turning the head quickly; had lancinating pains, worse at the menstrual period. Conium cured.

Also had greatly relieved prolapsus uteri, third degree, in an old woman, by giving Conium 70m., Fincke. The head symptoms ruled.

Dr. Finch attended a woman in confinement who had always suffered with mastitis after previous deliveries. This time he anticipated the trouble with Phytolacca, and there was nothing the matter with either breasts or nipples.

Adjourned.

EMMA D. WILCOX,

*Secretary.*

---

* An excellent indication for Hydrophobinum (or Lyssin, as it is now called) is: Can't drink water, but can drink milk, soups, wine, or warm drinks. A confirmation of this symptom was obtained by the editor in a case of croupous pneumonia.—ED.

